# Liquor licences issued to Australian schools

**DOI:** 10.1186/s12889-017-4613-0

**Published:** 2017-08-01

**Authors:** Bernadette M. Ward, Rebecca Kippen, Geoffrey Munro, Penny Buykx, Nyanda McBride, John Wiggers, Madeline Clark

**Affiliations:** 1 School of Rural Health, Monash University, PO Box 666, Bendigo, VIC 3552 Australia; 2 Alcohol and Drug Foundation, Melbourne, Australia; 3 School of Health and Related Research, University of Sheffield, Sheffield, UK; 4 National Drug Research Institute, Curtin University, Perth, Australia; 5 School of Medicine and Public Health, University of Newcastle, Newcastle, Australia

**Keywords:** Alcohol, Schools, Children

## Abstract

**Background:**

Children’s positive socialisation to alcohol is associated with early initiation of drinking and alcohol-related harm in adult life. Internationally, there have been reports of adults’ alcohol consumption at school events in the presence of children. The aim of this research was to identify the conditions under which Australian schools are required to apply for a liquor licence and the associated prevalence of liquor licences for these events where children were likely to be present.

**Methods:**

A document review was conducted to examine temporary liquor licensing legislation. Quantitative analysis was used to examine relevant licensing data. Coding criteria was developed to determine school type, student year levels and the likely presence of children.

**Results:**

Four jurisdictions provided data on 1817 relevant licences. The average annual licences/100 schools was highest amongst Independent schools followed by Catholic and public (government) schools. The rates were highest in Queensland and Victoria where children were present at 61% and 32% of events respectively.

**Conclusions:**

While there are legislative differences across jurisdictions, the prevalence of adults’ alcohol use at school events in the presence of children may reflect the various education department policies and principals’ and school communities’ beliefs and attitudes. Licences are not required for all events where liquor is consumed so the prevalence of adults’ use of alcohol at school events is likely to be higher than our analyses imply. Such practices may undermine teaching about alcohol use in the school curriculum and health promotion efforts to develop alcohol-free events when children are present.

## Background

Early initiation to drinking and risky use of alcohol by children and adolescents is associated with significant harm and alcohol dependence in adult life [1, 2]. Parents play a critical role in guiding their children’s perceptions and use of alcohol. For example, parental approval and use of alcohol is positively associated with children’s susceptibility to alcohol use initiation [3]. Schools also have a role in teaching young people about alcohol. This is generally embedded in curriculum [4], however, it is likely that schools can also influence alcohol perceptions and possibly use when adults consume alcohol at school events in the presence of children.

There have been media reports of adults consuming alcohol, when children are present, at school events in Australia and England and from the United States (US) [5–7]. The US and English reports have been described as school fundraising events (such as fetes or fairs), sporting days and discos. However, scientific publications in the area are limited. Two Australian studies of principals from New South Wales (NSW) and Victorian public (government) secondary schools (children aged 12–19 years) found that enrolled students and other children were present at school events when adults were consuming alcohol [8, 9]. These events occurred both on and off school premises and included graduations, debutante balls, fundraising, sporting/musical events and welcome barbeques [8, 9]. Principals’ support for these practices were positively associated with adults’ use of alcohol at such events [8]. Similarly, parents’ who agreed with such practices reported significantly higher alcohol consumption scores than parents who disagreed [10].

There is strong evidence for the positive association between alcohol availability and consumption [11]. One evidence-based strategy for reducing alcohol availability is the use of licensing [11]. Liquor licensing is used in 142 countries to determine the conditions under which alcohol may be sold and supplied [12]. In Australia, licensing requirements differ across states and territories [13]. The conditions under which alcohol is available at Australian school events, whether held onsite or elsewhere, are subject to both state/territory liquor licensing laws and education department policies. The latter may reflect the principles of legislation but vary widely and can be difficult to interpret [14]. Education department policies apply to public schools only and the existence and content of such policies in the Catholic and Independent school sectors is not known [14]. 

Licensing requirements for events held on school premises vary across Australian jurisdictions. The licence type is that of one-off community event categories [15–22]. A review of relevant education department policies on this issue found large variations [14]. No written policy exists in Queensland while in Victoria, much of the decision-making responsibility about adults’ use of alcohol at school events is devolved to the principal and school council [23, 24]. In contrast, the NSW the policy is very explicit; the consumption of alcohol at any school event (either on or off school premises) when any school children are present is not permitted [25].

Little is known about the conditions under which alcohol is available at school functions either on or off school premises. The aim of this research was to identify and compare the legal conditions under which Australian State and Territory Catholic, Independent and public primary and secondary schools, attended by children aged 4–19 years of age, are required to apply for a liquor licence and the associated prevalence of liquor licences granted to schools during 2012–14. In particular, we sought to identify the proportion of licences granted for events where children were likely to be present. We sought to identify if there was any relationship between liquor licensing conditions and the prevalence of liquor licenses issued to schools.

## Methods

### Study design

A document review was used to identify the circumstances under which Australian schools are required to obtain liquor licences, and a quantitative analysis of secondary data was used to determine the prevalence of them doing so.

### Data sources

In February 2015, liquor licensing legislation (Acts (including Amendments) and Regulations) from eight jurisdictions (NSW, Victoria, Queensland, Western Australia (WA), South Australia (SA), Tasmania, Northern Territory (NT), Australian Capital Territory (ACT)) were accessed via the internet. Representatives from the associated government departments in each State and Territory were contacted via telephone to verify these were current versions.

Raw data for all liquor licences issued to educational settings for a recent twelve month period were requested from seven jurisdictions (NT was excluded). In the absence of a specific identifier of licences issued to schools with students under the age of 18, department representatives were asked to filter their database to provide details of licences that included any of the following: “primary/secondary/junior/senior/school”. The details requested included location (postcode), title of the event, type/name of school and duration of licence. Both quantitative and qualitative data that provided information about licences granted to schools was requested. Except for one jurisdiction, data were provided free of charge. In NSW data were provided at a cost of AU$50/month of data access.

### Data cleaning and analysis

State and Territory liquor licensing legislation was reviewed and summarised under the following headings: relevant liquor legislation, title of the licence applicable for school related events, circumstances when a licence is required, any associated exemptions and/or restrictions, and the cost of applications.

Numerical and text licensing data was provided in an Excel spreadsheet. Data cleaning included removing temporary licences issued to community (e.g. street markets) and educational settings attended by adults only (e.g. university colleges). Except in NSW, less than 10% of records were excluded: (Victoria 65/639 (10%); Queensland 28/1120 (3%); Western Australia (WA) 0/128). The NSW records were unable to be filtered by the requested criteria and so included permanent (i.e. commercial > one occasion) licences. Of the 222,526 NSW records 22,503 (>99.9%) were excluded. After data cleaning, all fields were converted into quantitative variables to enable comparisons. Using the school names and internet confirmation, schools were coded by sector (Catholic, Independent, public: based on predominant funding source) and year level (primary: students aged 4–13 years, secondary: students aged 11–19 years, or combined primary/secondary schools: students aged 4–19 years). Schools were coded into metropolitan, regional and remote locations based on the Australian Standard Geographical Classification system [26]. This enabled geographical comparisons of licensing within jurisdictions. In the absence of quantitative data, qualitative text was used to identify the presence of children/students at licence events. Specifically, events such as fetes/fairs/fiestas, musicals/concerts/productions, presentations, beginning/end of year BBQ, art/fashion shows or events that were held outdoors were coded as having children/students present. To enhance coding reliability, 10% of records from each data set were independently coded by two researchers and differences were discussed until consensus was reached.

Data were analysed using descriptive statistics. The total number of schools across each sector were obtained from the Australian Bureau of Statistics [27] and differ by jurisdiction.

### Ethics

Approval for secondary data analysis was provided by Monash University Human Research Ethics Committee (2014–5650-5479).

## Results

The current and recent conditions under which Australian schools, attended by children aged 4–19 years of age, are required to apply for a liquor licence and the associated prevalence of licences granted during 2012–2014 varied across jurisdictions.

### Licensing legislation

As per Table 1, the title, circumstances, exemptions, restrictions and cost of liquor licences that apply to school based applications varied across time and jurisdictions. Historically, all jurisdictions required schools to apply for a licence when alcohol was sold. Amendments to the legislation in WA (2011), Queensland (2013) and NSW (2015) means a licence is no longer required for the sale of alcohol at many school functions (e.g. ‘small occasional functions’ (WA) and fundraising events (Queensland and NSW) (Table 1). The permissiveness or otherwise of licensing conditions (i.e. restrictions and requirements) differ across jurisdictions in relation to the number of one-off events covered by a single licence, the primary purpose of the event, responsible service of alcohol (RSA) certification requirements, type of containers to be used, number of bars and their opening hours. Similarly, the financial costs associated with gaining a licence vary from AU$20 in the Northern Territory to AU$107 in WA (assuming attendance is limited to 500 people).

**Table 1:** Licensing legislation (Acts and Regulations) and requirements, that apply to schools, for each Australian state and territory^a,b^

STATE / TERRITORY	New South Wales	Victoria	Queensland	Western Australia
Relevant Liquor Legislation	Liquor Act 2007 [16] and Regulations 2008 [39]	Liquor Control Reform Act 1998 [17] and Regulations 2009 [40]	Liquor Act 1992 [18] and Regulations 2002 [43]	Liquor Control Act 1988 [21] and Liquor Control Regulations [42]
Title of permit/licence applicable for school-related events	Limited licence[16] (s 36, [39] r 3)	Temporary limited licence[17] (s 14)	Community liquor permit[18] (s 103C)	Occasional liquor licence[21] (s 59)
Circumstances when licence/permit required	Sale or supply of alcohol at functions [16] (ss 36(1)(a)(ii), (2))	Alcohol sold for immediate consumption or as a fundraiser [17] (ss 14, 26)	Sale and consumption of alcohol at events run by ‘community organisations’ on ‘temporary or one-off basis’.Since 2013, only applicants for ‘high risk’ events are required to describe the license setting and provide an event management plan. [18] (ss 107B–D, [43])	Sale of alcohol at a function, or series of functions, over period up to 21 days, generally for immediate consumption.Applies also to provision of alcohol paid for by ticket or entry fee. [21] (s 59(1))
Exemptions from requirement for permit (that applies to some of the data used in this research study)^b^	Since March 2015, non-profit organisations may hold up to 6 ‘fundraising’ functions per year without permit. [16] (s 6(5), [44])	Alcohol provided free of charge or raffled.[17] (s 136(1), [45])	Since 1 July 2013, permits no longer required for ‘fundraising’ events run for the benefit of ‘the community’, including raffles in which liquor is ‘part of the prize’.[18] (ss 13–14, [43])	Since 16 July 2011, licence no longer required for ‘small occasional functions’ (fewer than 100 attending - served for up to 2 h - or fewer than 75 - served for up to 4 h) provided they start after 6 am and finish by 10 pm. [42] (r 8A), [46].
Restrictions associated with permit/licence	Alcohol consumed on premises, sold in open containers. For exempt (fundraising) functions, food and free water must be provided, single bar operating, service limited to 4 h. [16] (s 6(5), 38(1))	Maximum of 6 one-off events or ‘season’ of three months or 12 market stalls in a year.[17] (ss 14(1)–(1B))	For exempt functions, at which consumption of alcohol is ‘ancillary’ to purpose, must be sold in open containers, over 8 h only (not before 7 am or after midnight).[18] (s 13(b)-(c))	Service of alcohol must be ‘ancillary’ to purpose of the event. All events must be supervised by a certified ‘Approved Manager’ and all bar staff must hold a RSA certificate.[42] (r 8A).
Cost of permit/licence $ (at August 2015)	Single function - $80 online/ $150 paper-based. Multi-function - $500 application and $100 base annual fee thereafter plus variable charge.[39] (Sch 1)	$56.80 (if no other liquor licence held - $105.30 otherwise).[40] (Pt 5)	$62.20 per day (unless exempt).[41] (Sch1)	Varies according to numbers attending: $107 for up to 500 people, $219 for 501–1000, $1098 for up to 5000, $2197 for 5001–10,000, $4401 for over 10,000 people.[42] (Sch 3)
STATE / TERRITORY	South Australia	Tasmania	Northern Territory	Australian Capital Territory
Primary Liquor Legislation	Liquor Licensing Act 1997 [22] and Regulations [47]	Liquor Licensing Act 1990 [22] and Regulations [48]	Liquor Act 2012 [20]	Liquor Act 2010 [19] and Fee Detemination 2016 [49]
Title of permit/licence applicable for school-related events	Limited licence - sale of liquor[22] (s 41)	Special permit[22] (s 15)	Special Licence and ‘Continuing Special Licence’[20] (s 57)	Liquor permit (non-commercial) [19] (ss 47, 49)
Circumstances when licence/permit required	Sale or supply of alcohol at a ‘special occasion’ or series of such occasions (of 1–30 days duration, but authority may extend ‘in special circumstances’).[22] (s 41(1), (2))	Sale of alcohol on a one-off or intermittent basis, at functions in unlicensed premises, including where just an entry fee is involved [22] (ss 3, 15)	Sale or supply of alcohol at functions run by community organisations.	Sale of alcohol for consumption on or off premises (by non-profit organisation), according to permit - may be related to designated (possibly recurring) event [19] (s 49)
Exemptions from requirement for permit	Alcohol served free of charge, provided this is not attached to cover charge, meal cost or ‘donation’. Won in raffle etc. [22] (ss 4, 29, 129)	Alcohol provided free of charge or BYO, or as component of hamper not consumed on premises. [22] (ss 3, 5)	Private functions where alcohol served free of charge [20] (s 4)	Private function with fewer than 30 attending [19] (s 12(2))
Restrictions associated with permit/licence	Repeated requests for a limited licence may lead to denial of such a licence and diversion to another form of licence.[22] (s 41(5))	Sale of alcohol must be ancillary to purpose of event [48]	Alcohol consumed on permitted premises. Where functions involve minors, only wine and low-alcohol beer, and service restricted to two hours in late afternoon (conditions at the discretion of the D-G and Minister).[20] (ss 59, 59A)	
Cost of permit/licence $ (at August 2015)	$79 per function per day, but may be waived if function is held for charitable or community benefit, extending beyond members of organisation itself. [47] (Sch 3)	Four categories: Annual permit ($392.60), 6 month permit ($196.30), permit valid for 4–30 days ($166.10), permit valid for less than 4 days ($60.40) [48] (Sch 1)	$20 for Special Licence, Continuing Special Licence and renewal of Special Licence (no published regulation stipulating fees) - https://nt.gov.au/industry/hospitality/liquor-licence-fees)	$45 (retail value of liquor sales less than $2070) or $159 (liquor sales over $2070) [49] (Sch 1)

^a^The table includes corresponding sections (s/ss), rules (r), parts (Pts) and schedules (Sch) of the primary and subordinate legislation to which the requirements refer

^b^In the last five years, there have been changes to liquor licensing legislation in NSW, Queensland and WA. The data in this study precedes the changes in NSW and Queensland but not in WA where the legislation changed in 2011

### Licences issued

As per Table 2, four States (NSW, Victoria, Queensland, WA) provided data on 1817 relevant licences. The annual rate of licences issued/100 Catholic, Independent and public schools was highest in Queensland and lowest in NSW. The rates in Queensland were more than twice those in Victoria, five times those in WA and sixty times those in NSW.

**Table 2:** Average annual liquor licences per 100 schools and annual licence totals, by type of school and year level; percentage distributions of length of licence and whether children present at events for which licence was held—selected Australian states

State Year	New South Wales 2014	Victoria 2013	Queensland 2012	Western Australia 2014	Total
	Average annual licences per 100 schools^a^ (annual licence totals in parentheses)				
Type of school					
Catholic	1.5 (9)	30.0 (146)	133.7 (393)	21.6 (35)	38.1 (583)
Independent	1.8 (6)	65.0 (134)	115.4 (210)	62.6 (87)	50.9 (437)
Public	0.4 (8)	19.3 (294)	39.5 (489)	0.8 (6)	14.0 (797)
Year levels					
Primary	0.4 (9)	23.0 (357)	50.3 (580)	6.5 (44)	18.0 (990)
Secondary	1.6 (8)	21.1 (71)	73.5 (186)	1.9 (2)	22.1 (267)
Combined	1.3 (6)	44.1 (146)	105.5 (326)	27.5 (82)	40.1 (560)
State Total	0.7 (23)	25.9 (574)	63.7 (1,092)	11.9 (128)	22.5 (1,817)
Length of licence	Percentage distribution				
1 day	Not available	87.2	93.3	72.7	
2-14 days		4.9	5.9	25.0	
More than 14 days		7.8	0.8	2.3	
Total		100.0	100.0	100.0	
Children present^b^	Percentage distribution				
Yes	Not available	31.5	60.9	Not	
No		68.5	39.1	available	
Total		100.0	100.0		

a. 100*(Total number of licences)/(Total number of schools). Number of schools in New South Wales 2014, Victoria 2013, Queensland 2012 and Western Australia 2014 sourced from Australian Bureau of Statistics 2015. *Schools, Australia, 2014*, catalogue number 4221.0,\Canberra. The ‘Combined’ year level includes P–12 with pre-schools, kindergartens and early-learning centres, and all special schools.

b. Victoria: outside events assumed to have children present; Queensland: coding of qualitative text.

The average annual licences/100 schools was highest amongst Independent schools followed by Catholic and public schools. This was consistent with the weighted average results across all jurisdictions. In Victoria, the Independent school sector had the highest rate of accessing liquor licences followed by the Catholic and public sector schools. By contrast, in Queensland and WA, the Catholic and Independent school sectors had comparatively high rates of liquor licences. In Queensland, the rates of liquor licences issued to Catholic and Independent schools was 89 and 64 times higher than in NSW, 6.2 and 1.8 times higher than in WA and 4.5 and 1.8 times higher those in Victoria.

The weighted average rate of licences granted/100 schools was lowest amongst primary schools, followed by secondary schools and highest amongst combined primary/secondary schools. In Queensland and Victoria there were variations in permits by school year level, with combined primary and secondary schools (primary to year 12, ages 4–19 years) most likely to obtain licences. However, in Queensland a higher proportion of secondary schools (students aged 11–19 years) obtained licences than primary schools (students aged 4–13 years), whereas in Victoria a similar proportion of primary and secondary schools obtained licences. In WA and Victoria, a low proportion of primary schools, and similar proportion of secondary and combined schools obtained licences. Within jurisdictions there was no difference in the rate of licensing between metropolitan, regional or remote locations.

The vast majority of licences were issued for one day. Victorian schools were slightly more likely to apply for licences of more than 14 days duration. WA schools, although having a lower rate of total licences, were more likely to receive licences that were between 2 and 14 days duration. Children were present at 32% of the Victorian and 61% of the Queensland school events.

## Discussion

This is the first review of liquor licensing legislation and the associated granting of licenses for school events where children might be present. The findings demonstrate legislative variation and practices across jurisdictions. While the results may reflect differences in the ease of obtaining a licence or relative permissiveness of licensing requirements, there is no clear relationship between liquor legislation and the prevalence of licences issued for school events where children may be present.

Licensing legislation alone does not appear to explain the relatively high annual licensing rates in Queensland and Victoria and in contrast, the relatively low rates in NSW. Instead, the clarity of education department policies across jurisdictions might explain our findings. The relatively high rates of annual licences issued to Queensland schools may reflect the lack of education department policy [14]. Similarly, the relatively high rates of annual rates of licences issued to Victorian schools may reflect the devolution of decision making to Victorian school principals and councils [14, 23]. Equally, the restrictive policy of the NSW education department is consistent with the relatively low rates of licences issued to NSW schools [14, 25]. Liquor licensing provides the legislative framework within which jurisdictional education departments and individual schools must operate regarding alcohol at school events. The liberalisation of liquor licensing laws may provide few impediments to the consumption of alcohol at school events where children are present so there is a need for explicit education department policies to guide school staff and communities.

Independent and Catholic schools are not bound by government education department policy on this issue. However, the trend within and across jurisdictions is consistent; the annual rates of licences issued to Independent and Catholic schools reflect or is higher than those in public schools. The reason for this is unclear. It may be linked to the informal adoption of education department policies by Independent and Catholic schools. Alternatively, it may reflect underlying alcohol norms within school communities. Qualitative interviews with Victorian public and Catholic secondary school principals suggest there are a range of views regarding the acceptability of adults’ use of alcohol within school communities and sectors [8]. Reviews of Catholic and Independent schools’ policy on this issue need to highlight these differences and ensure their practices are consistent with ensuring the sectors uphold teaching about alcohol and adopt a health promotion approach by developing alcohol-free events when children are present.

There have been some reports of alcohol-related violence at school events [28] but little is known about potential long-term benefits or harms for children associated with alcohol use at primary and secondary school events (on or off premises). Currently, there are Australian guidelines recommending alcohol not be used in school fundraising events [29]. However, this is not reflected in the liquor licensing laws and there is no national government-endorsed strategic document which specifically addresses the use of alcohol at school events when children are present. The guidelines may be more accessible if they were incorporated into the relevant sector education department policies and national guidelines for low-risk drinking.

The increased social marketing of alcohol products means that children are exposed to alcohol at many social events [30, 31]. While the prohibition of alcohol at all events is not acceptable [11], the World Health Organization (WHO) supports the development of alcohol-free environments particularly when children people are present [32]. School communities, parents and peers can influence children’s initiation and use of alcohol [33–36] so the normalisation of alcohol at school events may undermine messages about alcohol in the school curriculum and efforts to develop alcohol-free environments when children are present. Adults who support drinking at school events in the presence of children are significantly more likely to have higher alcohol consumption scores [10] so there is a need to support school principals/councils/boards/parents about the potential harms associated with these practices.

Our reports of the use of alcohol at school events, when children are present, are likely to be conservative. The analysis only applies to the granting of licences for the sale of alcohol and so does not include events where applications were not made or approved. Nor does the data account for occasions when a licence is not required [ie. alcohol is provided free of charge and/or brought to the school (bring your own – BYO)] by event attendees. These events remain largely unrecorded and undescribed [8]. Similarly our data does not include school events that were held off-site at a licensed premise with a permanent licence. This may explain our finding of no difference in licensing rates by school location. Rates of alcohol consumption in rural and remote Australia are consistently higher than those in metropolitan areas [37]. The frequency of off-site functions, BYO and/or supplied alcohol at school events in rural areas may exceed that which is accounted for by licensing data.

This descriptive study had limitations. By design, it cannot determine causality. Licensing data were not available for all Australian States and Territories and there was some variation in the timeframes (2012–14) of the data provided by jurisdiction. Our analysis predates changes in NSW and Queensland legislation allowing “small occasional functions” in schools without a liquor licence. However, this is not the case for WA, so licensing data for that state is unlikely to capture the full scale of school alcohol-events. We excluded the NT because of the additional alcohol restrictions in remote Indigenous communities [20] and were unable to adjust our estimates to exclude schools in the many remote communities in WA which have introduced additional similar alcohol controls [21]. These restrictions may result in an underestimation of the rate of licences granted to those schools in communities where alcohol is allowed.

While there are different licensing requirements across jurisdictions, greater consistency between data recording formats for temporary licensing would assist in the ongoing monitoring and potential impact of changes in licensing legislation, not only for school events, but for a range of settings when children are present. In light of the ongoing liberalisation of liquor licensing, further research is needed to examine the impact of interventions aimed at supporting school communities to use evidence-based approaches when using alcohol at school events.

## Conclusions

To our knowledge, this is the first study to examine the requirements for Australian schools to obtain a liquor licence and to use liquor licensing data to quantify the prevalence of events at which alcohol is served in schools in the presence of children. Australia has a high level of alcohol use by global standards [32], and within the Australian context alcohol produces a high burden of disease and associated costs [38]. The results of this study suggest there are inconsistencies in liquor licensing requirements and the use of alcohol at school events across jurisdictions. Current practices are not consistent with WHO recommendations regarding alcohol-free environments when children are present. In the last decade, the circumstances in which Australian schools are required to apply for a liquor licence so that alcohol can be provided/consumed in the presence of children have been reduced in some jurisdictions. This means there is an important role for education department policy and school communities to educate, monitor and evaluate the use of alcohol at school events; particularly when children are present.

## Abbreviations

BYO: bring your own
NSW: New South Wales
US: United States
WA: Western Australia
WHO: World Health Organization
